# Supplementary figures and images for: Phylogeography of Arabidopsis halleri (Brassicaceae) in mountain regions of Central Europe inferred from cpDNA variation and ecological niche modelling
Source: PeerJ. 2016 Jan 28;4:e1645. doi: 10.7717/peerj.1645 (PMC4734066; doi:10.7717/peerj.1645)

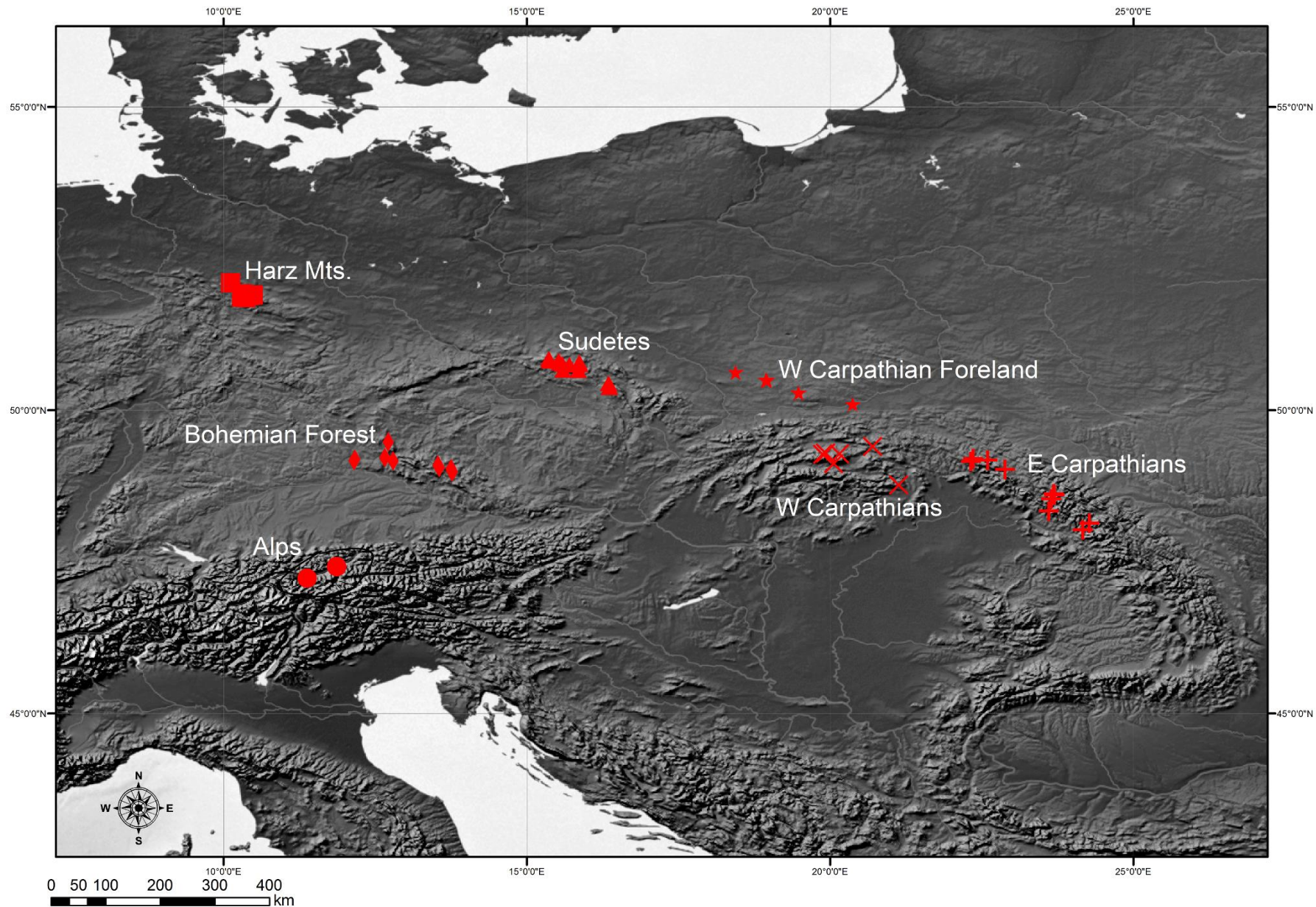

Supplement: Figure S1 [file peerj-04-1645-s001.pdf]

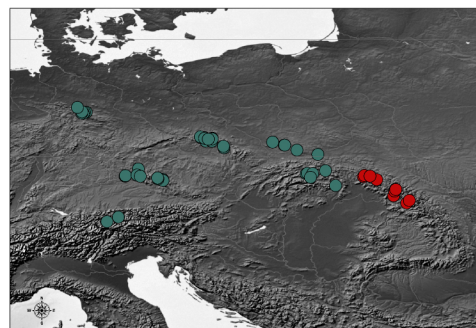

k=2

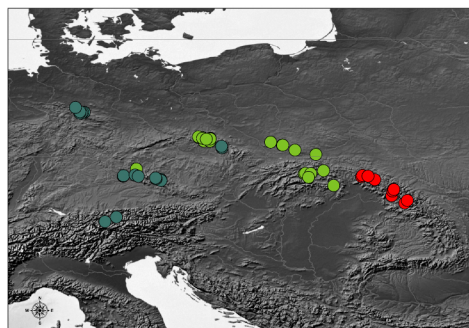

k=3

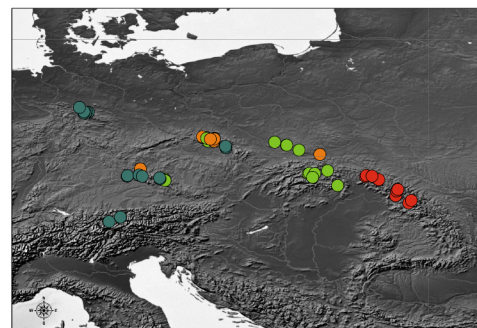

k=4

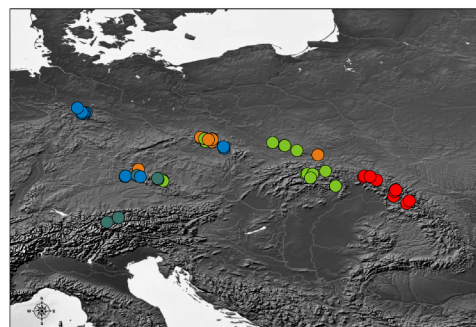

k=5

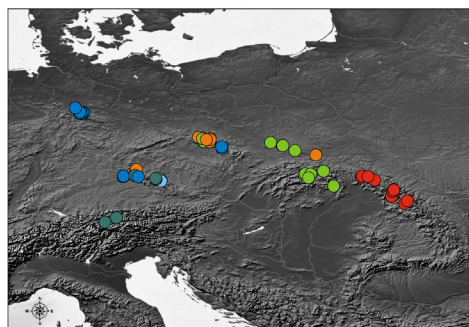

k=6

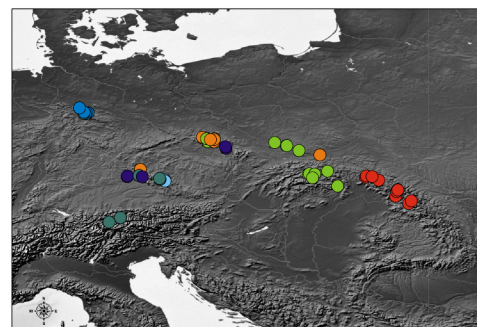

k=7

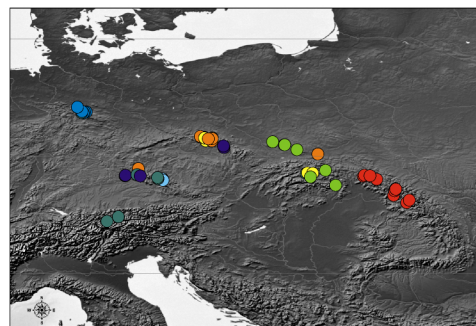

k=8

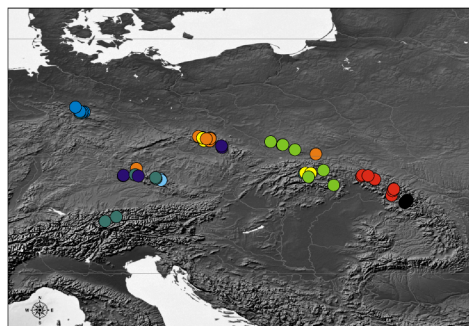

k=9

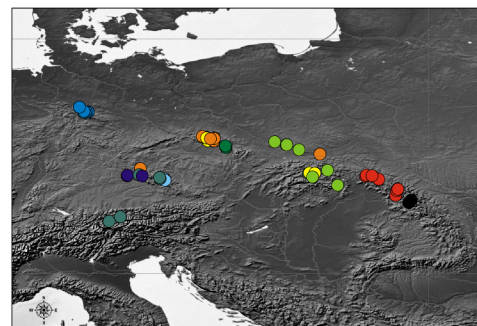

k=10

Supplement: Figure S2 — SKMC of investigated populations carried out using Shperikm (Hill, Harrower & Preston, 2013). Different levels of data structure are presented from k = 2 (two groups) to k = 10 (ten groups of populations). Population differentiation was inferred from a data matrix of chlorotype frequencies. [file peerj-04-1645-s005.pdf]
