# Supplementary material for: Phylogeography of Arabidopsis halleri (Brassicaceae) in mountain regions of Central Europe inferred from cpDNA variation and ecological niche modelling
Source: PeerJ. 2016 Jan 28;4:e1645. doi: 10.7717/peerj.1645 (PMC4734066; doi:10.7717/peerj.1645)
Supplement: Table S1 [file peerj-04-1645-s002.pdf]

S1 Table. Primer sequenes used to obtain analysed cpDNA fragments

assay	fragment	primer sequence (5'→ 3')
CAPS	*trn*C-*trn*D	F: CCAGTTCAAATCCGGGTGCC
		R: GGGATTGTAGTTCAATTGGT
SNaPshot	*trn*C-*trn*D	F: CCAGTTCAAATCCGGGTGCC
		R: GGGATTGTAGTTCAATTGGT
SNaPshot	*psb*C-*trn*S	F: GGTCGTGACCAAGAAACCAC
		R: GTTTCGAATCCCTCTCTCTC
SNaPshot	*trn*K1-*trn*K2	F: GGGTTGCCCGGGACTCGAAC
		R: CAACGGTAGAGTACTCGGCTTTTA
length difference	*trn*C-*trn*D	FM13: CACGACGTTGTAAAACGACTGTTTTCGTTTTCTAGACGTTCG
		R: TTGGATTTATGTTTTATTGACTCATTT
length difference	*trn*K1-*trn*K2	FM13: CACGACGTTGTAAAACGACTTGCACACAGCTTTCTCTATGT
		R: GACCCCATAAATGAAATCGAAA
