# Supplementary material for: Phylogeography of Arabidopsis halleri (Brassicaceae) in mountain regions of Central Europe inferred from cpDNA variation and ecological niche modelling
Source: PeerJ. 2016 Jan 28;4:e1645. doi: 10.7717/peerj.1645 (PMC4734066; doi:10.7717/peerj.1645)
Supplement: Table S2 [file peerj-04-1645-s003.pdf]

S2 Table. Primer sequences used in SNaPshot assay.

**primer**	**sequence (5'→ 3')**
SNPCS205F	ACTTCCCCACCTAGC
SNPCS1516F	AGCCAATAAAGATAAAAATA
SNPCD427R	GGGGGGAGTATCCAAGAAATCT
SNPmatK1609F	CCCCCCACTGGTATGCCCCAAAAAGG
SNPmatK1493F	CCCCCCCCCCCCTGTAAATTATAAGATTTTTT
SNPmatK2455F	CCCCCCCCCCCCAAAATAAGGTTGTACCGATC
SNPmatK368F2	CCCCCCCCCCCCCCCCCCATTATTCATGATTGACTAAA
SNPmatK2049R	CCCCCCCCCCCCCCCCCCCCATCTTGTTTTGTTATTCTAT
SNPmatK584F	CCCCCCCCCCCCCCCCCCCCCCCCCCCCCCCAAAGACGAAGTATATATTT
